# Editorial: The heme oxygenase system in immunity

**DOI:** 10.3389/fimmu.2025.1740080

**Published:** 2025-11-19

**Authors:** Diego L. Costa, Emanuela M. Bruscia, Dean Willis

**Affiliations:** 1 Departmento de Imunologia, Instituto de Ciencias Biomedicas, Universidade de Sao Paulo, São Paulo, Brazil; 2 Department of Pediatrics, Yale University School of Medicine, New Haven, CT, United States; 3 Department of Neurosciences, Physiology & Pharmacology, University College London, London, United Kingdom

**Keywords:** heme oxygenase, iron, carbon monoxide, biliverdin, bilirubin, innateimmune system, adaptive immune system, inflammation

Heme oxygenase (HO) is an enzyme that catalyzes the rate limiting step in the catabolism of heme, which results in the release of equimolar amounts of ferrous iron, the gas carbon monoxide and the bile pigment biliverdin which is subsequently converted to bilirubin by the enzyme biliverdin reductase (1).

Two functional isoforms of the enzyme are found in humans, being HO-1 the most studied and well characterized of them. While HO-2 is expressed mainly in testes, gastrointestinal tract and the brain during homeostasis (2), HO-1 expression can be induced in most cells of the human body, usually in response to oxidative but also other types of stresses (3). HO-1 is highly expressed in spleen and liver macrophages, where it plays a major role in recycling iron from heme contained in hemoglobin molecules released by phagocytosed senescent erythrocytes (4). The accumulation of heme as well as the activation of macrophages by a number of inflammatory mediators induces the expression of HO-1 in these cells (5). The enzyme detoxifies heme, which has important toxic oxidant effects in cells, but the products of heme degradation by HO-1 themselves also display potent antioxidant activities and protect cells from damage caused by oxidative stress. In addition to that, the expression and activity of HO-1 promote immunomodulatory effects in macrophages, such as suppression of pro-inflammatory cytokine production (6). Immunomodulatory activity of HO-1 has also been demonstrated in other leukocyte populations, such as dendritic cells, where the enzyme promotes decrease in antigen presentation capacity and production of pro-inflammatory cytokines (7, 8), and CD4^+^ T lymphocytes, in which HO-1 promotes immunosuppressive regulatory T cell activities and impairs Th1 and Th17 effector functions (9).

Due to its important immunomodulatory properties, the HO-system has attracted considerable attention as a potential therapeutic target by which to treat several pathological conditions be they infectious, inflammatory or neoplastic in nature. This Research Topic has gathered four articles, including one mini-review and one review, along with two original articles.

The pivotal study by Schuster et al. explores the critical function of HO-1 in regulating interactions between endothelial cells and monocytes, with significant implications for transplant vasculopathy (TV). Transplant vasculopathy remains a major challenge in organ transplantation, primarily driven by donor-specific antibodies that trigger inflammatory responses and vascular injury. This study highlights the importance of HO-1 in attenuating these harmful responses and mitigating TV.

Specifically, the authors demonstrate in an *in vitro* co-culture model that stimulation of endothelial cells with anti-HLA-I antibodies increases monocyte adhesion and transmigration via the adhesion molecule E-selectin (CD62E), which is expressed on cytokine-activated endothelial cells. This process, however, is significantly reduced by pharmacological induction of HO-1. In a murine heterotopic aortic transplantation model, modulation of HO-1 reduced TV, suggesting a protective role against graft inflammation and intimal hyperplasia. Thus, targeting HO-1 provides a novel therapeutic approach to prevent TV and enhance graft survival, offering a promising strategy for improving post-transplant outcomes. Beyond these findings, the study also underscores the broader range of biological mechanisms that rely on the HO-1 pathway to maintain immune responses in check, revealing new avenues for therapeutic intervention (10).

High extracellular free heme levels are associated with several pathologies including sickle cell anemia, malaria and sepsis. It is suggested that in these situations free heme causes tissue damage via generating reactive oxygen species, interfering with the functions of lipid membranes and/or acting as a DAMP to activate immune cells. Increased cellular HO-1 expression is believed to be protective against this damage caused by free heme (5). The paper by Nakagiri et al. reports on the use hemopexin, a heme scavenging protein, as a therapeutic strategy to prevent tissue damage caused by extracellular free heme. Using a mouse pulmonary ischemic-reperfusion model of lung injury, Nakagiri et al. demonstrate that hemopexin administration decreased tissue damage, coagulation and markers of inflammation. Interestingly, hemopexin also decreased HO-1 expression in the ischemic lungs, suggesting that the tissue damaging effect of extracellular heme had been decreased by hemopexin. Being an endogenous high affinity protein, hemopexin has some advantages as a therapeutic e.g. biological starting point, favorable PK/PD and low immunogenicity; however further investigations will need to be carried out to validate its potential, in particular the therapeutic window for hemopexin administration for various pathologies will need to be established. It will also be interesting to see if hemopexin and HO-1 based therapies can be combined.

In the review by Yeudall et al. the authors discuss the role of heme as an important player in the pathogenesis of several inflammatory conditions and the critical role played by HO-1 expression in macrophages as means of attenuating it. More specifically, the authors discuss studies that demonstrate a detrimental role for heme accumulation and release in hemolytic diseases, inflammatory disorders, such as sepsis, and cardiometabolic diseases, such as atherosclerosis, in which heme promotes oxidative stress and inflammatory damage. In such scenarios, HO-1 activity in macrophages plays an important protective effect, by degrading heme, which per se results in reduction of oxidative stress and inflammation, but also by releasing the heme degradation products CO and biliverdin, further converted into bilirubin, which possess potent antioxidant and cytoprotective effects. However, besides in most cases heme is detrimental and HO-1 activity is beneficial, in some infectious diseases as well as inflammatory disorders, depending on the pathogen and on the redox state of host cells, the release of iron as a result of heme degradation by HO-1 can have detrimental effects, by inducing pathogen growth and necrotic cell death by ferroptosis, which promote inflammatory damage.

Finally, in the mini-review by O’Rourke et al., the authors discuss the importance of the NRF2-HO-1 axis in the regulation of the low-grade sterile chronic inflammation that develops in aged people, a phenomenon called “inflammaging”. Several factors are involved in the induction of this condition, however, the increase in ROS levels commonly found in aged people is a major player in the enhancement of pro-inflammatory mediator production associated with inflammaging (10). NRF2 works as both a sensor of oxidative stress and a transcription factor that activates the transcription of antioxidant effectors in cells, among which, is HO-1. In homeostasis, NRF2 is constantly ubiquitinated for proteasomal degradation by the action of KEAP1, whereas, in the presence of ROS, KEAP1 is modified and the ubiquitination of NRF2 ceases, allowing it to migrate to the nucleus and promote gene transcription (11, 12). The authors discuss studies in which NRF2-HO-1 pathway, along with products of heme degradation by HO-1 were demonstrated to have an important role in controlling oxidative stress-driven inflammation in diseases often associated with aging, such as neurodegenerative disorders, as well as cardiovascular and autoimmune diseases. They also discuss the potential use of activators of NRF2 pathway and HO-1 transcription, as well as KEAP1 inhibitors to treat such disorders, by citing studies in which these treatment strategies were employed (O’Rourke et al.).

In conclusion, we believe that the manuscripts contained in this Research Topic bring important new data as well as thoughtful insights on the role of the heme-oxygenase system in the modulation of immunity and inflammation and about how it can be targeted for the development of novel treatment strategies for a number of diseases.
